# A Two‐Sample Mendelian Randomization Research Examining the Causal Association Between Ischemic Stroke and Obstructive Sleep Apnea

**DOI:** 10.1002/brb3.70787

**Published:** 2025-08-22

**Authors:** Qing Yu, Wei Wei, Ying Yuan

**Affiliations:** ^1^ Department of Acupuncture and Neurology, Wangjing Hospital China Academy of Chinese Medical Sciences Beijing People's Republic of China; ^2^ Department of Geriatric, Wangjing Hospital China Academy of Chinese Medical Sciences Beijing People's Republic of China; ^3^ Department of Acupuncture and Neurology, Wangjing Hospital China Academy of Chinese Medical Sciences Beijing People's Republic of China

**Keywords:** cardioembolic stroke, ischemic stroke, Mendelian randomization, obstructive sleep apnea

## Abstract

**Background:**

Although prior observational research has indicated a potential association between ischemic stroke (IS) and obstructive sleep apnea (OSA), it is still unclear how the two conditions are causally related. The purpose of this study is to evaluate the causal association between IS, including large artery stroke (LAS), cardioembolic stroke (CES), small vessel stroke (SVS), and OSA by the Mendelian randomization (MR) approach.

**Methods:**

To avoid bias caused by heterogeneity among populations with different genetic backgrounds, this study selected only databases related to the European population for analysis. We adopted a two‐sample Mendelian randomization analysis to examine the causal relationship between IS (n = 440,328), LAS (n = 410,484), CES (n = 413,304), SVS (n = 198,048), and OSA (n = 451,618).The inverse variance‐weighted (IVW) method was used as the primary analysis method to assess causal relationships, with sensitivity analyses conducted using the leave‐one‐out method, MR‐Egger intercept test, MR‐PRESSO global test, and heterogeneity tests.

**Results:**

MR analysis results showed that the gene‐predicted CES was associated with a higher risk of OSA (IVW: OR = 1.07, 95% CI [1.02, 1.13], *p* = 0.004), but IS (IVW: OR = 1.05, 95% CI [0.96, 1.14], *p* = 0.268), LAS (IVW: OR = 1.01, 95% CI [0.97, 1.04], *p* = 0.736), and SVS (IVW: OR = 0.99, 95% CI [0.92, 1.08], *p* = 0.893) were not directly causally associated with the incidence of OSA. No level of heterogeneity was detected in the above results (*p* > 0.05), and sensitivity analysis also indicated no significant heterogeneity.

**Conclusion:**

Despite there is no direct causative link between OSA and IS, LAS, or SVS, this study indicates that CES is a risk factor for OSA and that CES and OSA are positively correlated at the genetic level.

## Graphical abstract

The present study employed a Mendelian randomization approach to evaluate the causal relationship between ischemic stroke (IS) and its three subtypes: large artery stroke (LAS), cardioembolic stroke (CES), and small vessel stroke (SVS), as well as obstructive sleep apnea (OSA). The present study found that CES was a risk factor for OSA, and there was a positive causal association between CES and OSA at the genetic level. However, there was no direct causal association between IS, LAS, SVS, and OSA.

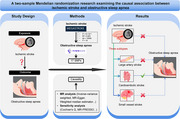

## 1 Introduction

Stroke is a common neurological disease, with ∼10 million people worldwide suffering from stroke each year (GBD 2019 Stroke Collaborators 2021). It is the second leading cause of death and a major cause of long‐term disability in humans (Katan and Luft 2018). Ischemic strokes (IS) are the most prevalent type of strokes, accounting for around 87% of all strokes (Benjamin et al. 2018). IS can be classified into five subtypes based on the underlying cause, namely cardioembolic stroke (CES), large artery stroke (LAS), small vessel stroke (SVS), stroke of other determined etiology (SOE), and stroke of undetermined etiology (SUE). Among these, the first three subtypes are the most common (Adams et al. 1993). IS can quickly result in neurological impairments such as hemiplegia and aphasia, which significantly lowers a person's quality of life and burdens the patient's family and society (Boot et al. 2020).

Obstructive sleep apnea (OSA) is the most common type of sleep apnea that produces recurrent apneas and hypopneas due to partial or complete collapse of the upper airway during sleep (Gottlieb and Punjabi 2020). In the general population, OSA affects 9%–38% of people, with 13%–33% of men and 6%–19% of women affected (Senaratna et al. 2017). Between 60% and 94% of obese patients fit the OSA diagnostic criteria (Loo et al. 2020). Research shows that OSA can directly cause intermittent hypoxia and hypercapnia, which in turn trigger sympathetic nervous system activation, oxidative stress, and systemic inflammation. Over time, this increases the risk of cardiovascular disease, hypertension, diabetes, and other conditions (Adderley et al. 2020). With OSA's incidence gradually increasing globally and the harm it causes to people's health increasing, identifying the direct risk factors is crucial to better preventing its onset (Correa et al. 2024).

Studies have indicated that around 70% of IS patients have OSA (Bassetti et al. 2020; Seiler et al. 2019), that OSA is closely associated with a greater rate of cardiovascular and stroke events in stroke patients, and that the mortality rate for OSA patients is higher than that of stroke patients without OSA (Alexiev et al. 2018). According to epidemiological studies, at least 50% of stroke patients have sleep‐related respiratory abnormalities, specifically OSA (Baillieul et al. 2022). Based on previous research, it can be preliminarily concluded that OSA is one of the risk factors for stroke, one of the complications after stroke, and stroke patients with OSA often indicate poor prognosis (Rola et al. 2008; Turkington et al. 2002). However, more large‐sample data research is still required to clarify whether stroke can be an independent risk factor.

We are well aware that confounding bias can affect observational studies, while Mendelian randomization (MR) effectively avoids these puzzles by using genetics to identify relationships between diseases and phenotypes. To evaluate the causal impact of the relationship between exposure factors and outcome events, it primarily employs single nucleotide polymorphisms (SNPs) as instrumental variables (IVs) for genetic variation (Burgess et al. 2019). These genetic variations satisfy the logic of the temporal sequence and are less vulnerable to reverse causality and confounding influences since they are inherited from the mother and assigned at random at conception (Davies et al. 2018). Other than randomized controlled trials (RCTs), these are now the clinical research methodologies with higher quality evidence.

The question, what are the pathogenic factors between OSA and IS, has been the subject of much discussion among academics (Xiaofen and Hongnian 2020). OSA and IS and its many subtypes do not appear to be causally connected, according to the findings of our early calculations and associated MR investigations (Li et al. 2023). Research on IS's potential role as a risk factor for OSA is currently insufficient, nevertheless. The small sample size and the interference of not examining the various IS subtypes separately may have impacted this study, producing biased results, even if some researchers stated in an MR study that there is no causal association between stroke and OSA (Cavailles et al. 2024). Finding the causes of OSA and implementing targeted early intervention can significantly lower medical costs, prevent and treat multi‐system damage in OSA patients at an early stage, and improve their quality of life. This is because of the growing body of research on the disease and its clinical harm. To attempt to find disease‐causing factors that are directly linked to OSA at the genetic level and to aid in the early detection and treatment of OSA in the clinic, this study further investigates the causal relationship between IS and its LAS, CES, and SVS subtypes and OSA using a two‐sample MR method.

## 2 Methods

### 2.1 Design of Study

**FIGURE 1:**
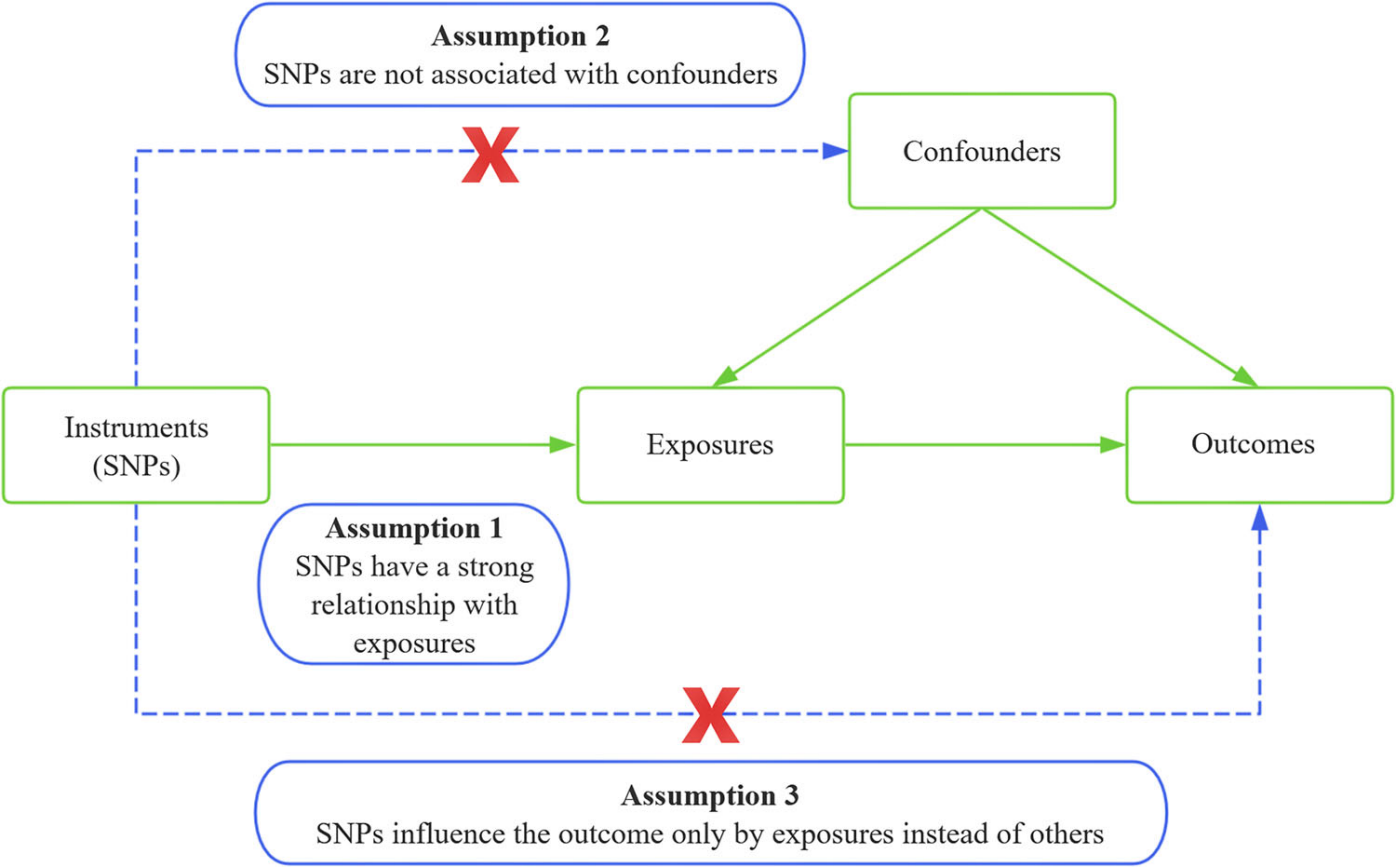
Study design diagram and three assumptions of Mendelian randomization. Abbreviation: SNPs, single nucleotide polymorphisms.

OSA was the outcome variable in our investigation, which employed SNPs linked to IS and its LAS, CES, and SVS subtypes as IVs. The MR analysis method was utilized to perform causal association analysis, and the heterogeneity of IVs was evaluated using Cochran's *Q* test. Sensitivity analysis was completed in the end. The three MR assumptions listed below are satisfied by this study: (Ference et al. 2021) I. The correlation assumption: exposures and IVs have a high association; II. The independence assumption: exposure‐outcome and IVs are not confounded; III. The exclusion premise states that the chosen genetic variation can only influence the result via the “IVs‐Exposures‐Outcome” path and cannot influence the outcome via any other path (Figure 1).

### 2.2 Data Sources

The FiNNGen consortium (Kurki et al. 2023) recently published a genome‐wide association study (GWAS) that provided the OSA data. About 451,618 Europeans participated in the study, comprising 401,418 controls and 50,200 OSA patients, for a total of 21,306,774 SNPs. The METASTROKE consortium (Malik et al. 2018) is the source of the IS dataset. This analysis solely used data from GWAS of IS about the European ethnic group to prevent bias brought on by genetic heterogeneity. The IS study covered 8,340,184 SNPs with 34,217 cases and 406,111 controls. The IS cohort primarily comprises three subtypes: LAS, CES, and SVS. About 4373 cases and 406,111 controls were included in the LAS analysis, which covered 8,451,005 SNPs; 7193 cases and 406,111 controls were included in the CES analysis, which covered 8,306,069 SNPs, and 5386 cases and 192,662 controls were included in the SVS analysis, which covered 876,5838 SNPs (Table 1).

**TABLE 1:** Detailed information on the GWAS included in Mendelian randomization analysis.

Exposures/outcomes	Consortium	PMID	Year	Cases/controls	nSNPs	Ethnicity
Obstructive sleep apnea	FiNNGen	—	2024	50,200/401,418	21,306,774	European
Ischemic stroke	MEGASTROKE	29,531,354	2018	34,217/406,111	8,340,184	European
Large artery stroke	MEGASTROKE	29,531,354	2018	4373/406,111	8,451,005	European
Cardioembolic stroke	MEGASTROKE	29,531,354	2018	7193/406,111	8,306,069	European
Small vessel stroke	MEGASTROKE	29,531,354	2018	5386/192,662	8,765,858	European

*Note*: Data are summarized from GWAS databases from IEU OpenGWAS databases (https://gwas.mrcieu.ac.uk/).

**Abbreviations: FINNGEN, Finnish Gene; nSNPs, number of single nucleotide polymorphisms**.

### 2.3 Selection of Instrument Variables

The following quality control procedures are used in our study to choose the appropriate IVs for MR analysis aiming to guarantee the validity and precision of the causal association between IS and its LAS, CES, and SVS subtypes and OSA. First, SNPs significantly associated with IS and its subtypes were selected as IVs, and SNPs with a threshold lower than the genome‐wide statistical significance threshold (5×10^−8^) were selected as IVs. Third, we evaluated the correlation between SNPs and exposure, eliminated SNPs with weak statistical power (*F* < 10), and evaluated the strength of the IVs by computing the F statistic, which is defined as *F* = β^2^ /SE^2^ (Feng et al. 2022). Fourth, project the SNPs associated with IS and its three main subtypes onto the GWAS pooled data for OSA and extract the corresponding statistical parameters. During this matching process, there may be cases where exposed SNPs cannot be matched in the outcome dataset. Considering that the absence of these SNPs may be due to sample size limitations or other technical issues in the outcome dataset, we chose not to seek proxy SNPs to ensure the robustness and reliability of the analysis results while avoiding potential biases introduced by the use of proxy SNPs. Fifth, the same effect allele was used to reconcile the effects of SNPs on exposure and outcome. To ensure consistency in the definition of effect alleles, we used dbSNP build 155 (based on the GRCh37/hg19 genome) as the sole standard: the reference allele (ref allele) defined in this database is the reference allele used in our analysis, and the corresponding non‐reference allele (alt allele) is the effect allele reported and analyzed in our study. To harmonize allele directions across the datasets involved in the study, we used the *harmonise_data()* function from the R package TwoSampleMR (v0.5.6) to automatically harmonize the two datasets. This function automatically identifies matching types by comparing allele frequencies and chain directions: if effect alleles are inconsistent, the effect values (β) are reversed and allele labels are swapped; chain‐ambiguous SNPs (e.g., A/T, C/G) are automatically excluded. We used the final SNPs for the MR analysis.

### 2.4 Methods of Statistics for Analysis

To evaluate the causal relationship between IS and its LAS, CES, and SVS subtypes and OSA, our study employed five methods: the MR‐Egger technique, weighted median estimator (WME), inverse‐variance weighted (IVW), simple mode (SM), and weighted mode (WM). The IVW method combines a meta‐analysis approach along with a Wald value estimate for each SNP to get an overall assessment of the impact of IS and its LAS, CES, and SVS subtypes on OSA, assuming that all genetic variations are legitimate IVs. The IVW result is regarded as impartial in the absence of horizontal pleiotropy (Bowden et al. 2016). The intercept term can be used to determine whether pleiotropy is present because the MR‐Egger approach is predicated on the idea that IV strength is independent of the direct effect (instrument strength independent of direct effect, or InSIDE). Horizontal pleiotropy is not present if the intercept term is equal to zero, and the MR‐Egger regression's findings are in line with IVW (Nazarzadeh et al. 2020). Even when more than 50% of IVs are erroneous, the WME technique can accurately estimate causality because it takes advantage of the intermediate effect of all genetic variation that is available (Zhao and Schooling 2021). The WME approach outperforms the MR‐Egger technique 24 in terms of test efficiency, bias, and type I error rate when the InSIDE assumption is broken. The test of the median, which combines SNPs with comparable causal effects and yields the causal effect estimate of the majority of clustered SNPs, is the foundation of both the SM and WM methods. According to studies (Z. Lin et al. 2021), the IVW method outperforms the other four MR test methods in terms of test efficiency. Therefore, the primary technique for estimating causal effects in this investigation was the IVW approach, which was also utilized to analyze the results. The study's beta values were transformed into odds ratios (OR) and 95% confidence intervals (CI) to aid in the interpretation of the results. The MR‐Egger intercept method and the MR‐PRESSO method were used to detect the presence of horizontal pleiotropy. The MR‐PRESSO method includes (1) a global test for horizontal pleiotropy, (2) outlier detection, and (3) distortion test after removing the detected outlier SNPs to assess the level of pleiotropy in the remaining SNPs. If outliers are detected, they are removed from the IVs, and the MR causal assessment is re‐performed (Verbanck et al. 2018). To evaluate potential bias in causal impact estimations resulting from measurement inaccuracy of SNPs brought on by various experimental circumstances and examined populations, Cochran's *Q* statistic was utilized to test for IV heterogeneity. We evaluated the impact of each SNP on the result by systematically disregarding each SNP in a one‐at‐a‐time study to find potentially heterogeneous SNPs. For MR analysis, this research used the “TwoSampleMR (v0.5.6)” software package and the R language program (v4.5.1).

## 3 Results

### 3.1 Selection of Instrumental Variables

This study used IS and its subtypes LAS, CES, and SVS as exposure factors, and OSA as the outcome factor. After excluding the two SNPs identified as abnormal by the MR‐PRESSO method (rs11242678, rs7766042), our study ultimately included 17 SNPs as IVs for analysis, of which six were associated with IS, three with LAS, four with CES, and four with SVS. We also excluded any SNPs with linkage disequilibrium, palindromic structures, or partial variants and maintained an intermediate allele frequency. All included SNPs had *F*‐values >10, indicating no weak instrumental bias in our study. Detailed information on these SNPs is provided in Tables S1–S5.

### 3.2 MR Analysis

**FIGURE 2:**
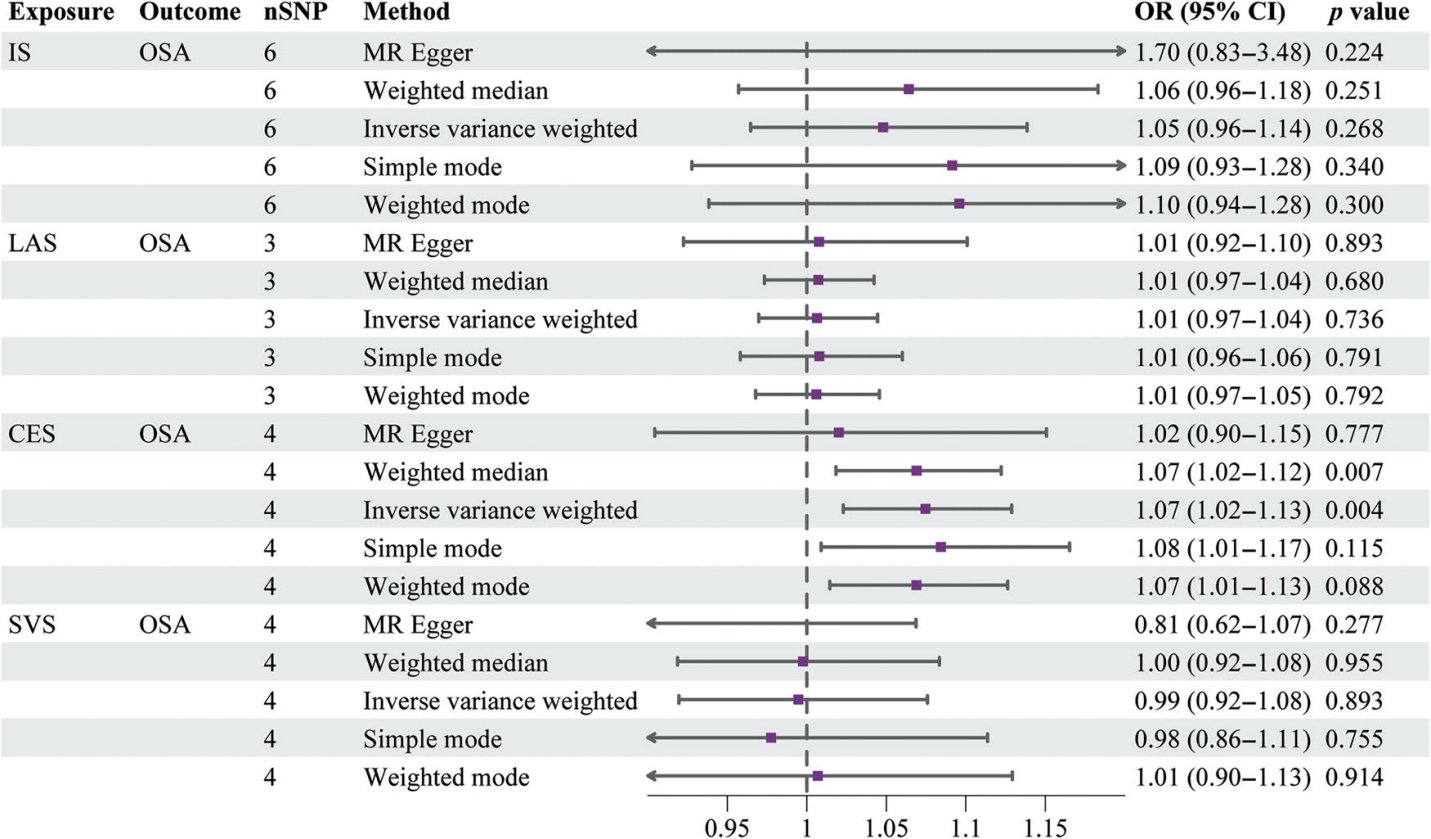
Mendelian randomization analysis of the causal effect. Abbreviations: CES, cardioembolic stroke; CI, confidence interval; IS, ischemic stroke; LAS, large artery stroke; nSNPs, number of single nucleotide polymorphisms; OR, odds ratio; OSA, obstructive sleep apnea; SVS, Small vessel stroke.

**FIGURE 3:**
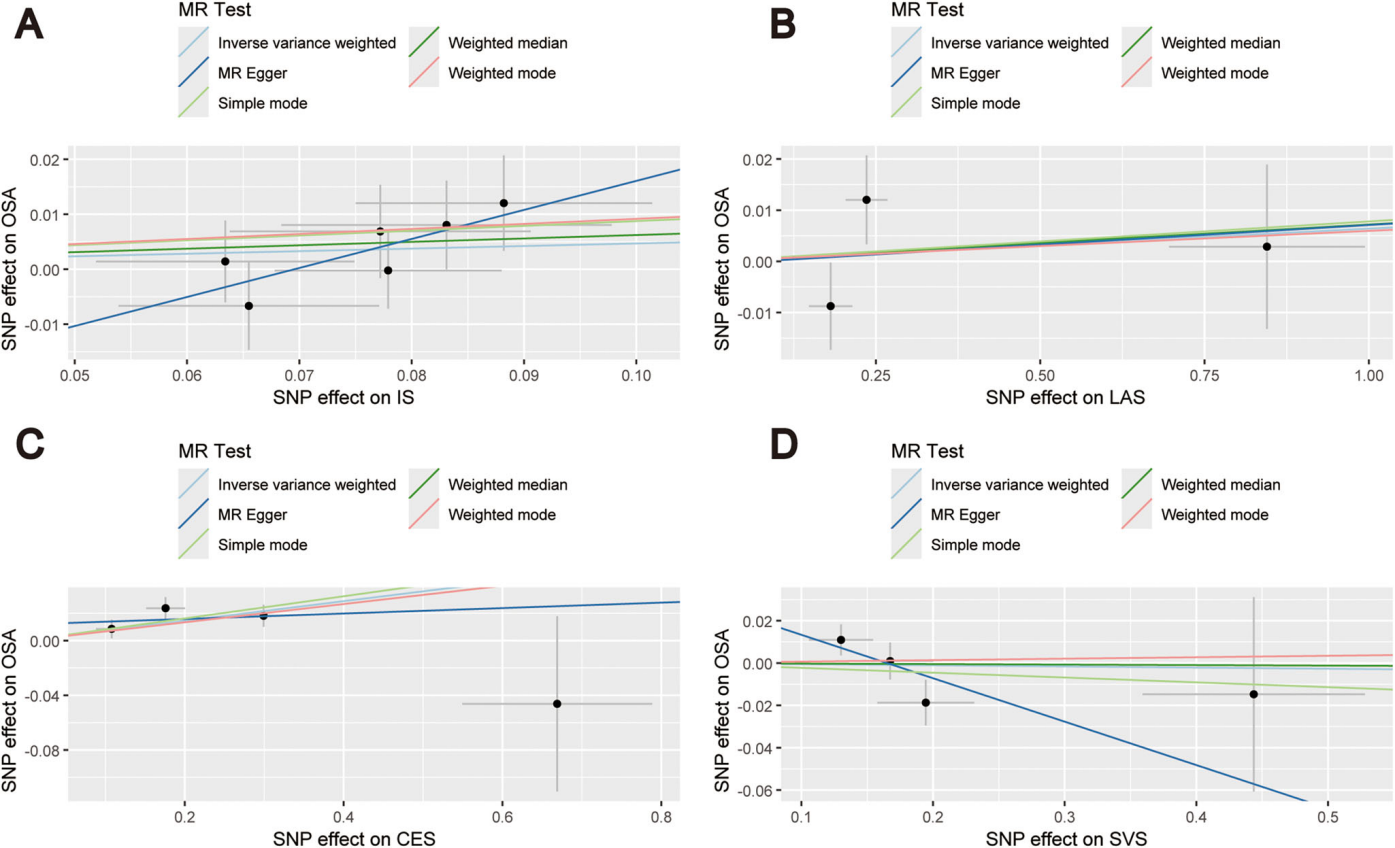
Mendelian randomization analysis scatter plots. (A) ischemic stroke; (B) large artery stroke; (C) cardioembolic stroke; (D) small vessel stroke. Abbreviations: CES, cardioembolic stroke; IS, ischemic stroke; LAS, large artery stroke; MR, Mendelian randomization; OSA, obstructive sleep apnea; SNP, single nucleotide polymorphism; SVS, small vessel stroke.

**FIGURE 4:**
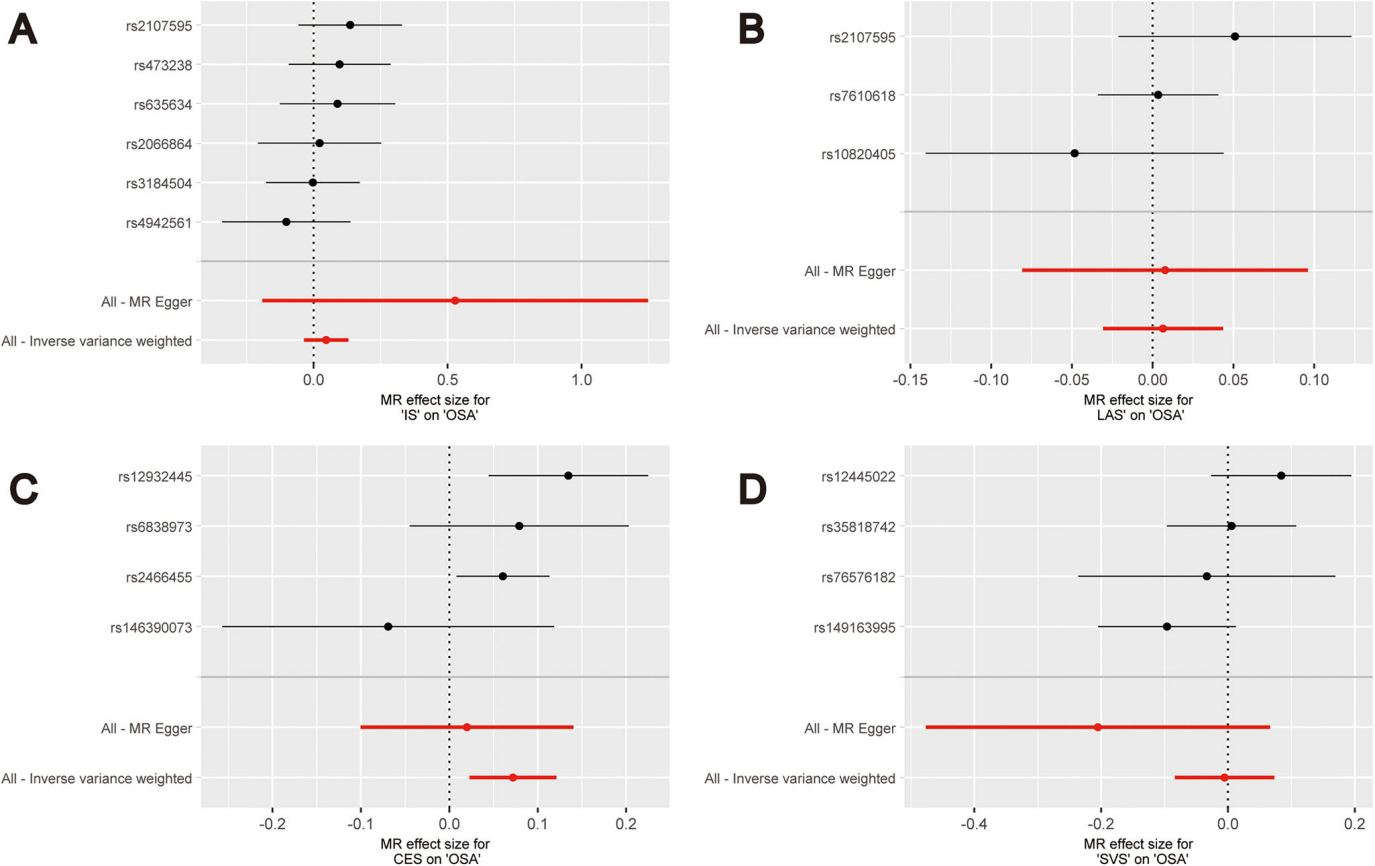
Forest plot of genetic associations. (A) ischemic stroke; (B) large artery stroke; (C) cardioembolic stroke; (D) small vessel stroke. Abbreviations: CES, cardioembolic stroke; IS, ischemic stroke; LAS, large artery stroke; MR, Mendelian randomization; OSA, obstructive sleep apnea; SVS, small vessel stroke.

Figure 2 displays the findings of the MR analysis for OSA, IS, and its LAS, CES, and SVS subtypes. Using the IVW method, we discovered that OSA was not directly caused by IS, LAS, or SVS, nor did these raise the incidence of OSA. The results of their analyses were as follows: IS (IVW: OR = 1.05, 95% CI [0.96, 1.14], *p* = 0.268), LAS (IVW: OR = 1.01, 95% CI [0.97, 1.04], *p* = 0.736), and SVS (IVW: OR = 0.99, 95% CI [0.92, 1.08], *p* = 0.893). CES subtypes and OSA incidence, however, had a strong positive causal association (IVW: OR = 1.07, 95% CI [1.02, 1.13], *p* = 0.004). The findings of the IVW study were in agreement with the findings of the analysis of the other four MR methods for IS and its LAS, CES, and SVS subtypes. The scatter plots and forest plots of the MR analysis of IS and its LAS, CES, and SVS subtypes on OSA are shown in Figures 3 and 4.

### 3.3 Sensitivity Analysis

**FIGURE 5:**
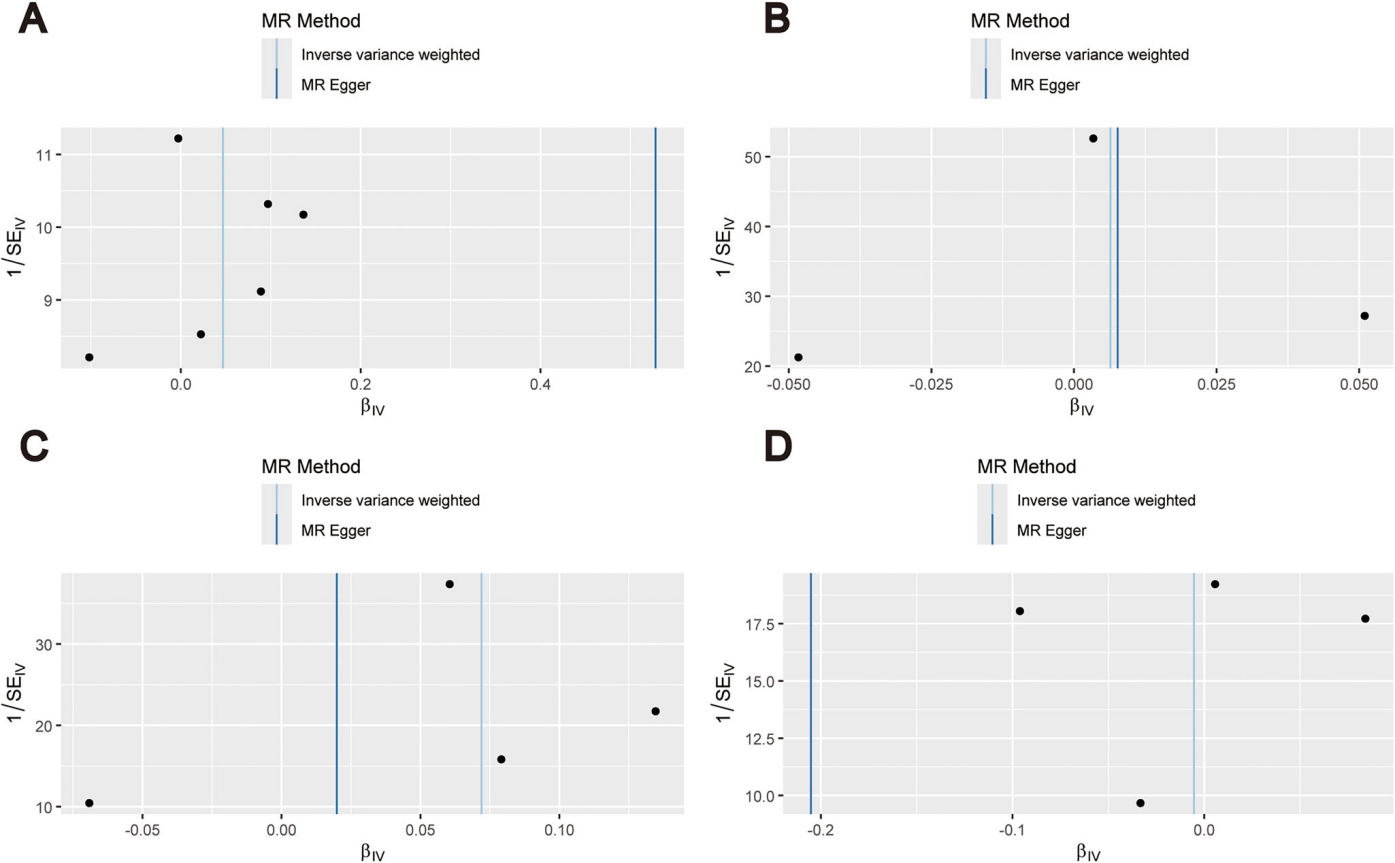
Scatter and funnel plots for each relationship. (A) ischemic stroke; (B) large artery stroke; (C) cardioembolic stroke; (D) small vessel stroke. Abbreviation: MR, Mendelian randomization.

**FIGURE 6:**
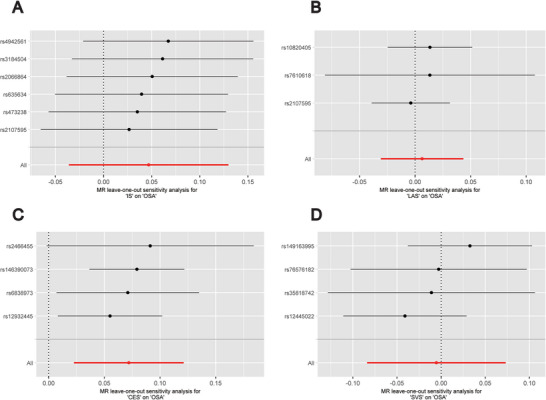
The leave‐one‐out‐sensitivity forest plot of genetic associations. (A) ischemic stroke; (B) large artery stroke; (C) cardioembolic stroke; (D) small vessel stroke. Abbreviations: CES, cardioembolic stroke; IS, ischemic stroke; LAS, large artery stroke; MR, Mendelian randomization; OSA, obstructive sleep apnea; SVS, small vessel stroke.

Table 2 shows the findings of the sensitivity analysis. The results of Cochran's *Q* test show that there is no significant heterogeneity in all results (*p* > 0.05), indicating that there is no heterogeneity between SNPs. In addition, the MR‐Egger regression intercept did not reveal any horizontal multieffectivity, and the results of the MR‐PRESSO global test also confirmed that there was no significant horizontal multieffectivity in our study, indicating that SNPs do not significantly affect OSA through pathways other than IS and its LAS, CES, and SVS subtypes. According to the leave‐one‐out technique test, no single SNP significantly alters the MR results, and the results are stable. The findings of this investigation are steady since the points on the left and right sides of the IVW line in the funnel plot are approximately symmetrical. Figures 5 and 6 display the funnel plots and leave‐one‐out method analysis plots for the MR analysis.

**TABLE 2:** Sensitivity analysis of the Mendelian randomization analysis results.

Exposure	Heterogeneity test		Pleiotropy test		MR‐PRESSO global test (*P*)
	Cochran's *Q* test	*p*	Egger intercept	*P*	
Ischemic stroke	3.087	0.687	−0.037	0.258	0.704
Large artery stroke	2.846	0.241	−0.001	0.978	NA
Cardioembolic stroke	4.223	0.238	0.012	0.450	0.418
Small vessel stroke	5.313	0.150	0.034	0.275	0.231

## 4 Discussion

This study explored the causal link between OSA and IS and its subtypes using MR analysis techniques and the GWAS databases that were already in place. To lessen the effect of confounding variables and guarantee the validity of the study results, the MR analysis was carried out using five methods: MR‐Egger, WME, IVW, SM, and WM. A strict sensitivity analysis was also conducted. The results of the study indicate that there is a significant causal relationship between CES and OSA, while no significant causal relationship was found between IS, LAS, SVS, and OSA. CES increases the risk of OSA, so awareness of secondary OSA in CES patients should be raised with the aim of early prevention of OSA.

The incidence of OSA is linked to stroke, according to a growing body of observational studies conducted in recent decades. Although a prior bidirectional MR study on the causal relationship between stroke and OSA demonstrated that there is no causal relationship between the two conditions, the results may have been impacted by the small sample size and lack of targeted analysis of each subtype of stroke in the stroke GWAS database (Cavailles et al. 2024). Large‐scale epidemiological studies have confirmed that sleep apnea is highly prevalent among patients with stroke or transient ischemic attack (TIA). A meta‐analysis (Hasan et al. 2021) involving 64,047 patients showed that the prevalence of severe sleep apnea (Apnea‐Hypopnea Index, (AHI), > 15) during the acute, subacute, and chronic phases of stroke was 31.6%, 36.1%, and 25.1%, respectively. According to research by Huhtakangas et al. (2017), two‐thirds of stroke patients suffer from moderate‐to‐severe sleep apnea, which is common among stroke patients in the acute phase. Sonja et al. (2021) also found that patients with ischemic stroke have a higher probability of developing sleep apnea, and the incidence of OSA is higher than that of central sleep apnea (CSA), further proving that OSA is more likely to develop after ischemic stroke. These observational studies' findings, however, are limited because they are unable to account for the influence of potential confounding variables, such as age, gender, and underlying medical conditions. Although our study did not discover a direct genetic connection between IS, LAS, SVS, and OSA, it does not rule out the possibility that these conditions could influence the development of OSA through other factors, including obesity, diabetes, and hypertension (Oscullo et al. 2019; Reutrakul and Mokhlesi 2017). In the future, it will be necessary to further prove the relationship between IS and OSA through a more comprehensive GWAS database.

In addition, our analysis revealed a strong causal link between CES and OSA, which is in line with the findings of other earlier observational studies (Bassetti et al. 2006; Young et al. 2008). At least 20% of IS patients are CES patients (Hart et al. 2014), and CES is more significant than other IS kinds. First, compared to other IS subtypes, cardiac embolism has a greater mortality rate and results in more severe and abrupt strokes (H. J. Lin et al. 1996). Second, in high‐income nations like Canada, a growing percentage of strokes are being caused by cardiac embolism as a result of better treatment for hypertension and dyslipidemia (Bogiatzi et al. 2014). Investigating the different risk variables following CES is therefore very important from a therapeutic standpoint. It is worth noting that due to the lack of direct measurement indicators of stroke severity during the acute phase in the underlying GWAS data (MEGASTROKE), we cannot completely rule out the possibility that the observed genetic risk of CES is associated with OSA, which may partially reflect the impact of more severe neurological dysfunction following this subtype of stroke rather than the uniqueness of the subtype itself. Future studies combining MR analysis with genetic tools for stroke severity will help further clarify this issue.

There are numerous advantages to our study. First, MR analysis offers a more effective way to investigate the etiology of complex diseases by avoiding reverse causality and confounding variables that are frequently present in observational studies. Second, to ensure the validity of the study findings, a sizable GWAS database was utilized to examine several IS cases and controls as well as their subtypes independently. Third, there is less chance of bias brought on by demographic stratification because every member of the sample is of European ancestry.

There are certain limitations to our research. First, the study population is restricted to the European population, mostly due to the relative amount of genomic databases and research data for this population. However, it is not possible to fully account for the relationship between the two diseases in other populations. Second, the MR approach ignores the influence of nonlinear interactions and is predicated on the idea that exposure and outcome have a linear relationship. Finally, Since the stroke dataset used in this study did not separately list the genetic data for the SOE and SUE subtypes, this study lacks MR analysis of the relationship between the SOE and SUE subtypes and OSA, which requires further refinement in future genetic studies.

## 5 Conclusion

In conclusion, this study concluded that there is no direct causal association between OSA and IS and its LAS and SVS subtypes using MR methods. CES is an independent risk factor for OSA, nonetheless, as evidenced by the positive causal link between CES and OSA at the genetic level. This study also has practical significance for the clinical prevention of OSA. We urge doctors to raise awareness of secondary OSA in CES patients. However, the specific mechanism of action of CES as a risk factor for OSA requires further investigation to find higher level evidence confirming the relationship between these two illnesses.

## Author Contributions

All authors contributed significantly to this research, gave final approval for the final version to be published, agreed to submit the article to the designated journal, and agreed to take responsibility for all aspects of this research. Specifically, Q.Y. was responsible for research design, implementation, article writing, and chart production. W.W. was responsible for data collection, interpretation, and analysis. Y.Y. was responsible for article revision and critical evaluation.

## Ethics Statement

This study utilizes aggregated data rather than individual‐level data. The data involved all originate from publicly published GWAS summary databases, which comply with the conditions for exemption from review as stated in the “Ethical Review Measures for Life Sciences and Medical Research Involving Humans.”

## Conflicts of Interest

The authors declare no conflicts of interest.

## Peer Review

The peer review history for this article is available at https://publons.com/publon/10.1002/brb3.70787.

## Supporting information



Table S1 MR analysis of ischemic stroke and obstructive sleep apnea SNPs Features
Table S2 MR analysis of large artery stroke and obstructive sleep apnea SNPs Features
Table S3 MR analysis of cardioembolic stroke and obstructive sleep apnea SNPs Features
Table S4 MR analysis of small vessel stroke and obstructive sleep apnea SNPs Features
Table S5 SNP characteristics of variants

## Data Availability

The data supporting the findings of this study are available on the IEU open GWAS.
